# Standardization of Multiparametric Prostate MR Imaging Using PI-RADS

**DOI:** 10.1155/2014/431680

**Published:** 2014-06-09

**Authors:** Joyce G. R. Bomers, Jelle O. Barentsz

**Affiliations:** Department of Radiology and Nuclear Medicine (766), Radboud University Medical Center, P.O. Box 9101, 6500 HB Nijmegen, The Netherlands

## Abstract

The purpose of this paper is to introduce and describe the Prostate Imaging and Reporting Archiving Data System (PI-RADS). For every single parameter the PI-RADS scoring system will be explained and magnetic resonance imaging (MRI) examples will be given. In the end two patient cases are presented to explain the overall interpretation score in multiparametric imaging.

## 1. Introduction

At present, multiparametric magnetic resonance imaging (MRI) is the most sensitive and specific imaging technique for localizing prostate cancer (PCa) [1]. According to a group of prostate MRI experts from the European Society of Urogenital Radiology (ESUR) multiparametric MRI should at least consist of high-resolution T2-weighted imaging (T2WI) in combination with two functional techniques, such as dynamic contrast-enhanced (DCE) MRI, diffusion-weighted imaging (DWI) or proton spectroscopy imaging (MRSI) [2]. The most important reason for this is that T2WI is the main important parameter to picture the prostate anatomy, and DCE-MRI adds sensitivity [3, 4] in PCa detection and DWI [5–7] and MRSI [8, 9] improve the specificity of PCa characterization.

Nevertheless, interpretation of multiparametric MR images is still subjective. For other organs, for example, the breast, liver, and thyroid gland, standardized interpretation schemes including risk stratification criteria have been developed to determine the presence of malignancy. The most developed and eminent system is the Breast Imaging Reporting and Data System (BI-RADS).

Inspired by BI-RADS and to improve the diagnostics of PCa, the same group of prostate MR imaging experts published in 2012 a set of clinical guidelines with the aim to standardize the interpretation and to report the different parametric MR-techniques: the Prostate Imaging and Reporting Archiving Data System (PI-RADS) [2]. Major goals of this system are to allow comparison of interobserver interpretation variability; to reduce this variability by discussing the individual scores; to enhance communication with clinicians in a uniform way; to facilitate quality assurance and research; and to improve patient outcome.

The purpose of this paper is to introduce and describe the PI-RADS system by means of MR imaging examples.

## 2. PI-RADS Scoring System

In the next sections the PI-RADS scoring criteria are explained for all parameters except MRSI. MRSI is, in contrast to T2WI, DWI, and DCE, considered as an optional parameter by the ESUR guidelines and is therefore not discussed in this report. Every parameter, T2WI, DWI, and DCE, is scored independently on a 5-point scale, where score 1 means that clinically significant disease is highly unlikely; score 2 means that clinically significant disease is unlikely to be present; score 3 indicates that the presence of clinically significant cancer is equivocal; score 4 means that clinically significant cancer is likely to be present; score 5 indicates that clinically significant cancer is highly likely to be present. Since the different parameters are not always unanimous in their scoring, the overall interpretation of each lesion is clarified in the end and a final score is given to predict its chance of being a significant cancer.

## 3. T2-Weighted Imaging

Overall, high-resolution T2-weighted imaging (T2WI) provides the best images to assess the anatomy of the prostate and its adjacent structures as the bladder, seminal vesicles, and rectal wall. In diagnosing prostate cancer it is a sensitive technique; however, it is not very specific.

From a radiological point of view the prostate consists out of two different zones: the peripheral zone, located posteriorly and inferiorly, and the central gland, located anteriorly and superiorly [10]. Anatomically, the central gland can be divided into the transition zone and the central zone. With increasing age, the composition of the central gland changes. In young men it is mainly composed of the central zone, but in older men it is mainly composed of transition zone, due to the development of benign hyperplasia (BPH), which leads to the formation of adenomatous nodules.

Given the distinct anatomical appearance of both zones on T2WI, two different sets of PI-RADS criteria are developed: one for the peripheral zone and another for the transition zone.

### 3.1. Peripheral Zone

**Figure 1:**
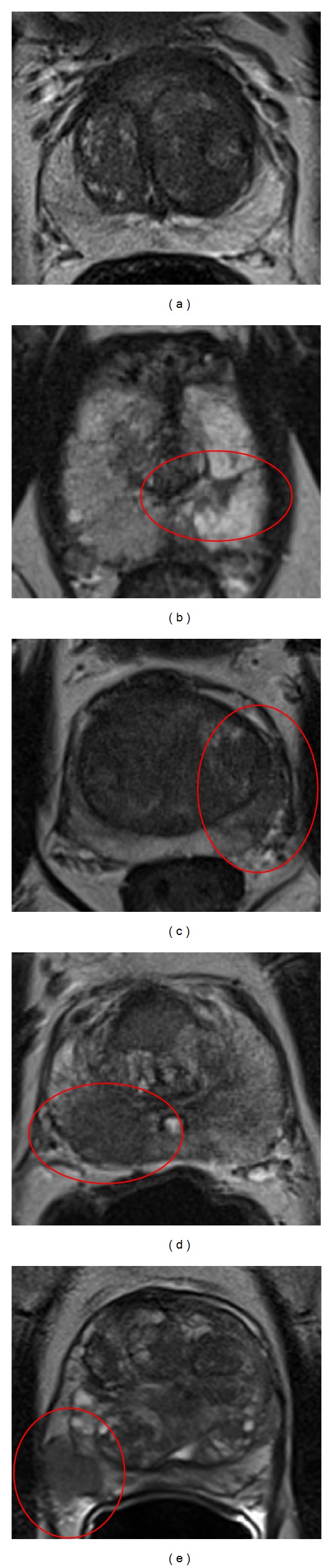
Examples of PI-RADS scoring for T2WI in the peripheral zone. (a) PI-RADS 1. (b) PI-RADS 2. (c) PI-RADS 3. (d) PI-RADS 4. (e) PI-RADS 5.

In Table 1 the PI-RADS criteria for the peripheral zone are shown. A healthy normal peripheral zone (PI-RADS 1) has a uniform high signal intensity as depicted in Figure 1(a). Linear, wedge-shaped, geographic areas of lower signal intensity with no clear delineation and no mass effect usually indicate nonmalignant conditions such as prostatitis, atrophy, scar tissue, hematomas, postradiation changes, or hormonal effects [11–13]. These lesions can be scored as PI-RADS 2.  Figure 1(b) shows a typical PI-RADS 2 lesion in the left peripheral zone because it is wedge-shaped and shows no mass effect.

Prostate cancer can appear as a focal area of low signal intensity, with decreasing signal intensity when the Gleason grade increases. A discrete, homogenous focus or mass with low signal intensity and still confined to the prostate is scored as PI-RADS 4 and is shown in the right peripheral zone in Figure 1(d). When this focus shows extracapsular extension or invasive behavior, mass effect on the capsule (bulging), or more than 1.5 cm capsule contact, it is highly likely to be clinically significant prostate cancer and should therefore be scored as PI-RADS 5. In Figure 1(e) a lesion with mass effect on the capsule and probable extracapsular extension is seen in the right peripheral zone.

A PI-RADS 3 score should be given when the lesion does not appear as described in the other categories. An example is given in Figure 1(c): the area in the left anterior horn of the peripheral zone is a PI-RADS 3 lesion because it is well demarcated and therefore cannot be scored as a PI-RADS 2, and as it does not show homogenous low signal intensity it cannot be scored as PI-RADS 4 as well.

### 3.2. Transition Zone

**Figure 2:**
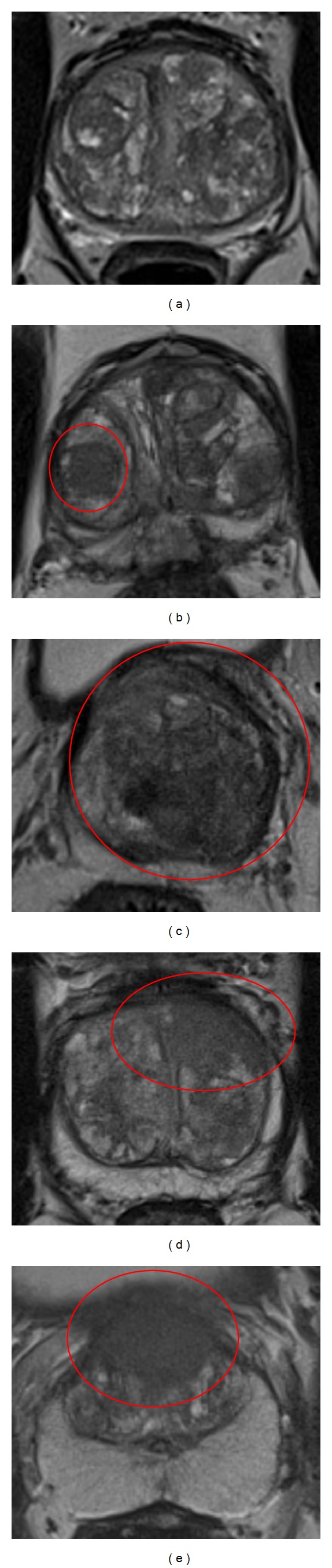
Examples of PI-RADS scoring for T2WI in the transition zone. (a) PI-RADS 1. (b) PI-RADS 2. (c) PI-RADS 3. (d) PI-RADS 4. (e) PI-RADS 5.

The PI-RADS criteria for scoring a lesion in the transition zone on T2WI are shown in Table 2. On T2WI a normal transition zone can be described as areas of low signal intensity alternated with round, well-defined BPH nodules with an inhomogeneous signal intensity, in a pattern described as “organized chaos” as depicted in Figure 2(a). The presence of clinical significant cancer is highly unlikely here; for this reason this can be scored as PI-RADS 1. When one or more of these areas show well-marginated homogenous signal intensity, for example, the indicated nodule on the right side in Figure 2(b), this should be scored as PI-RADS 2.

A focal, ill-defined area showing homogenous low signal intensity, also described as “erased charcoal drawing sign,” visible in Figure 2(d) in the ventral left part of the prostate, should be scored as PI-RADS 4. When this area involves the anterior fibromuscular stroma, extends into the anterior horn of the peripheral zone, and is lenticular or water-drop-shaped, it should be scored as PI-RADS 5. In Figure 2(e) the large lesion with erased charcoal drawing sign and involving the anterior fibromuscular stroma ventrally is a typical example of a PI-RADS 5 lesion. If the lesion does not fit in the criteria described above, it should be scored as PI-RADS 3. An example is given in Figure 2(c). This lesion in the left side of the prostate has a well-defined margin and could be scored as PI-RADS 2; however some erased charcoal effect is present, indicating PI-RADS 4. Because of the aforementioned reasons, it is neither a clear PI-RADS 2 nor a PI-RADS 4 lesion, and therefore scored as PI-RADS 3.

## 4. DWI

Diffusion-weighted imaging shows the random movement of water molecules in tissue. In tissues with high cellular density and intact cell membranes, for example, cancer or fibrosis, water molecules can hardly move. Then, the diffusion-weighted images will show diffusion restriction represented by a high signal intensity on the high *b*-value images and low signal intensity on the apparent diffusion coefficient (ADC) maps. ADC maps correlates well with tumor aggressiveness and therefore improves specificity in examining prostate MR images [5]. More technical details about diffusion-weighted imaging can be read in the report of Qayyum [14].

**Figure 3:**
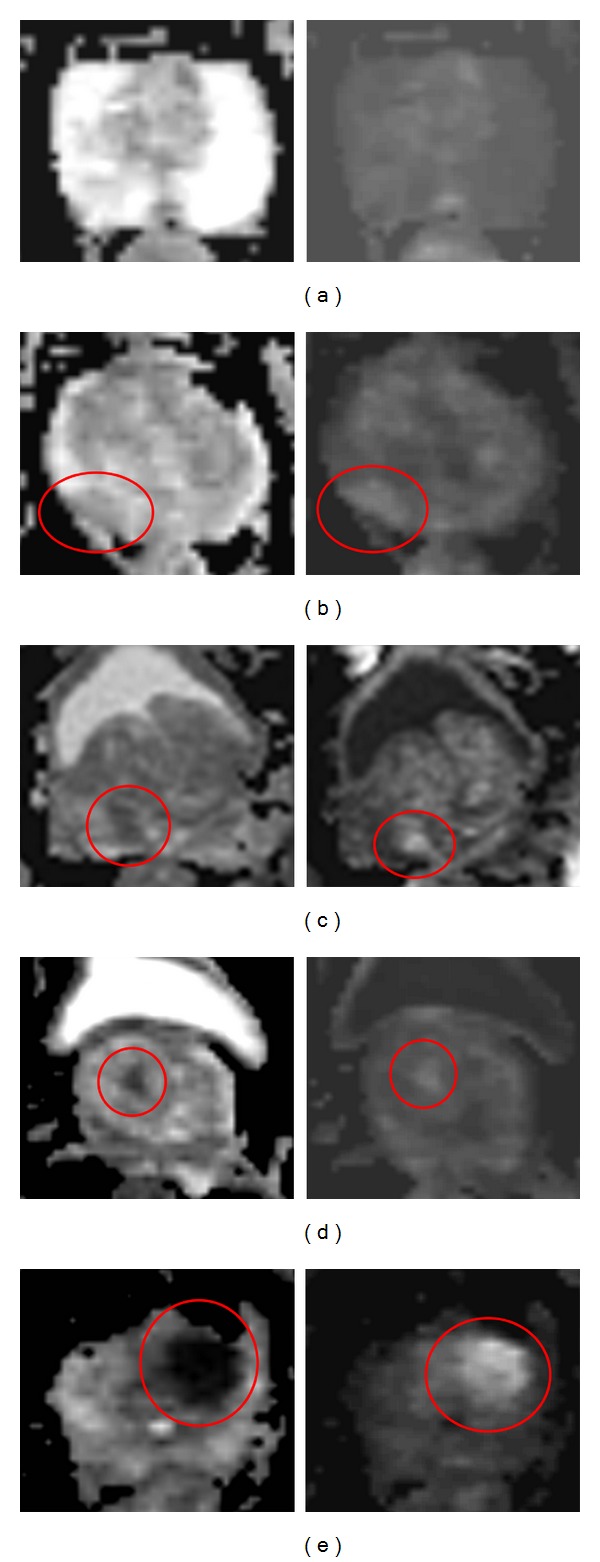
Examples of PI-RADS scoring for DWI. Left: axial ADC map. Right: axial DWI with calculated *b* = 1400. (a) PI-RADS 1. (b) PI-RADS 2. (c) PI-RADS 3. (d) PI-RADS 4. (e) PI-RADS 5.

Normal glandular prostate tissue (PI-RADS 1) will give no signal reduction on the ADC map and no increase in signal intensity on the high *b*-value images. An example is shown in Figure 3(a). On the left side the ADC-map is shown with particular in the peripheral zone high signal intensity and on the right side the calculated *b*1400 image shows equal signal intensity in the entire prostate.

A typical PI-RADS 2 lesion is represented by diffuse hypersignal intensity on high *b*-value images. Low ADC values; no focal features, however, linear, triangular or geographical elements are allowed. This is depicted in Figure 3(b), both the left and right peripheral zones show hypointense signal on the ADC map and diffuse high signal intensity on the calculated *b*1400 image.

When the ADC-map shows focal area(s) of reduced ADC together with isointense signal intensity on high *b*-value images, as depicted in the right transition zone in Figure 3(d), PI-RADS score 4 should be reported. A focal mass or area showing reduced ADC as well as hyperintense signal on the high *b*-value images is characteristic for PI-RADS 5. A typical example of a lesion with these characteristics is given in the left transition zone of the prostate in Figure 3(e).

If the diffusion-weighted images show intermediate appearances and no characteristics of categories 1/2 or 4/5, PI-RADS score 3 should be reported. In Figure 3(c) a PI-RADS 3 lesion is shown. On the ADC map no clear signal reduction is seen, whereas on the calculated *b*1400 image a focal lesion with high signal intensity is represented on the right dorsal side of the prostate. Based on the ADC map this lesion should be scored as PI-RADS 1; however based on the high *b*-value image the lesion should be scored as PI-RADS 5. Because it is neither a clear PI-RADS 1 lesion nor a clear PI-RADS 5 lesion, it was scored as PI-RADS 3. An overview of the scoring criteria is shown in Table 3.

## 5. DCE

Dynamic contrast-enhanced (DCE) MRI is based on the permeability of blood vessels and extravasation of contrast agent into the adjacent tissue. Leaky endothelia with an increased permeability and therefore fast contrast enhancement are a typical feature for PCa.

The PI-RADS scoring system for DCE imaging works different from the other parameters. These criteria are currently under discussion because according to some studies DCE-MRI does not seem to add significant value to the diagnosis of PCa [15, 16]. Other studies state that DCE-MRI can detect PCa with both high sensitivity and specificity and helps in tumor staging [17, 18]. Because of this debate, criteria will be modified almost certainly in the next PI-RADS version. Nevertheless, the current criteria for DCE-scoring are described in Table 4.

**Figure 4:**
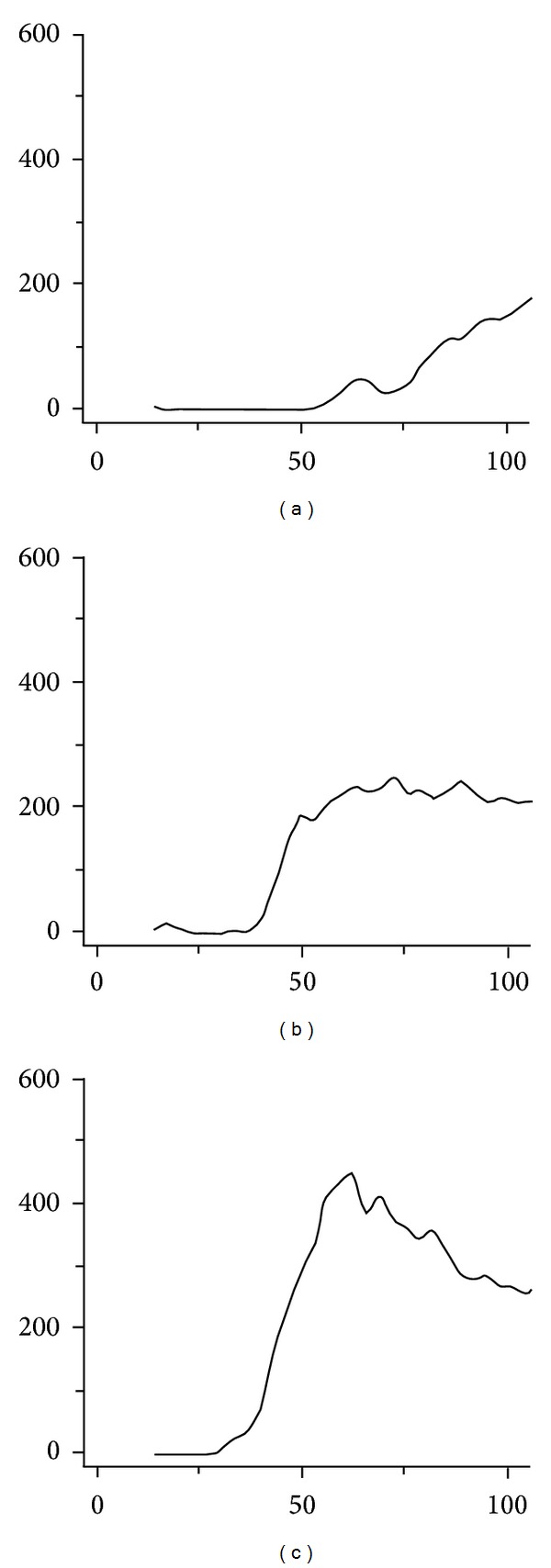
Examples of PI-RADS scoring for DCE. (a) Curve type 1. (b) Curve type 2. (c) Curve type 3.

The first step is to determine which signal-intensity-over-time curve type fits the enhancement pattern of the lesion. Typical examples of the different curve types (1, 2, and 3) are shown in Figure 4. If the curve type is known, the accompanying PI-RADS score should be given.

Curve type 1 can be described as slowly increasing enhancement over time, curve type 2 is characterized by enhancement reaching a plateau phase, and curve type 3 shows fast enhancement with washout effect afterwards.

When a curve type 2 or 3 is present, the second step is to verify whether the specific lesion is a focal enhancing lesion. If yes, one point should be added to the PI-RADS score. The third step is to determine whether the lesion is asymmetric or is located at an unusual place. If yes, again add another point to the PI-RADS score. In this way a maximum score of PI-RADS 5 can be achieved.

**Figure 5:**
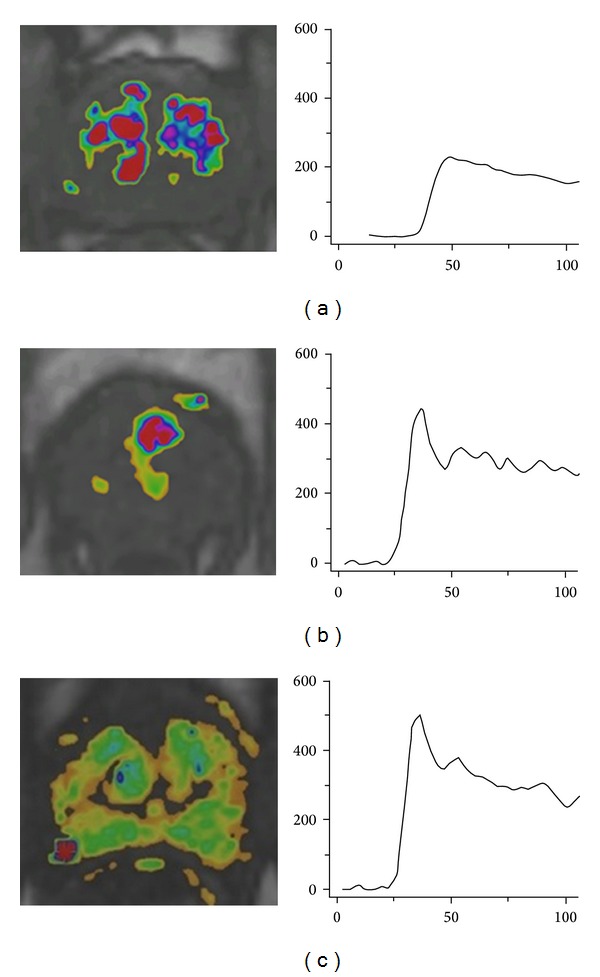
Examples of PI-RADS scoring for DCE. (a) PI-RADS 3. (b) PI-RADS 4. (c) PI-RADS 5.

In Figure 5(a) a DCE-image with the typical enhancement pattern of BPH is shown. The enhancement has a curve type 3, is not focal and not asymmetric, and is therefore scored as PI-RADS 3. In Figure 5(b) a prostate with an enhancing lesion in the ventral part of the prostate is shown. Again, a type 3 enhancement curve is shown. One point can be added to the PI-RADS score because it is a focal enhancing lesion, ending up with a final PI-RADS score of 4.  Figure 5(c) shows a focal, asymmetric enhancing lesion in the right peripheral zone with curve type 3 and can therefore be scored as PI-RADS 5.

## 6. Overall Interpretation

The ESUR guidelines provide explicit criteria for how to rate a lesion for every specific MRI parameter, but a consistent instruction on how to calculate the overall PI-RADS score is lacking. Since these guidelines were published, several research groups validated the PI-RADS score and most of them calculated a PI-RADS sum score (scale from 3 to 15) by summation of the 3 single scores [16, 19–24].

Another method to assign a final PI-RADS score is not to use the sum score of all different parameters, but rather an overall interpretation score, identical to the BI-RADS system. This means that the final score will not be in the range of 3–15, but in the range of 1–5.

The ESUR prostate MRI expert group and the PI-RADS steering committee of the American College of Radiology (ACR) recently have reached consensus how to classify this final PI-RADS score. In brief, it will contain the following: in case all individual parameters indicate the same level of suspicion for the presence of clinically significant Pca, for instance, T2WI, DWI, and DCE are all scored as PI-RADS 5, the overall PI-RADS score should be 5 as well. When not all parameters are consistent in determining the presence of clinically significant cancer, the new classification method prescribes that the “dominant” parameter should determine the overall PI-RADS score. The dominant parameter for cancer suspicious lesions in the peripheral zone is DWI, for transition zone lesions it is T2WI, and for lesions suspicious for PCa recurrence it is DCE. This method will be published in the second half of 2014 as modification to the current ESUR guidelines. Recently, part of this is also suggested by Baur et al. After evaluation of the current PI-RADS system, they concluded that assigning a DWI score for peripheral zone lesions and a T2WI score for transition zone lesions was sufficient for stratification of patients for further diagnostic workup [16].

**Figure 6:**
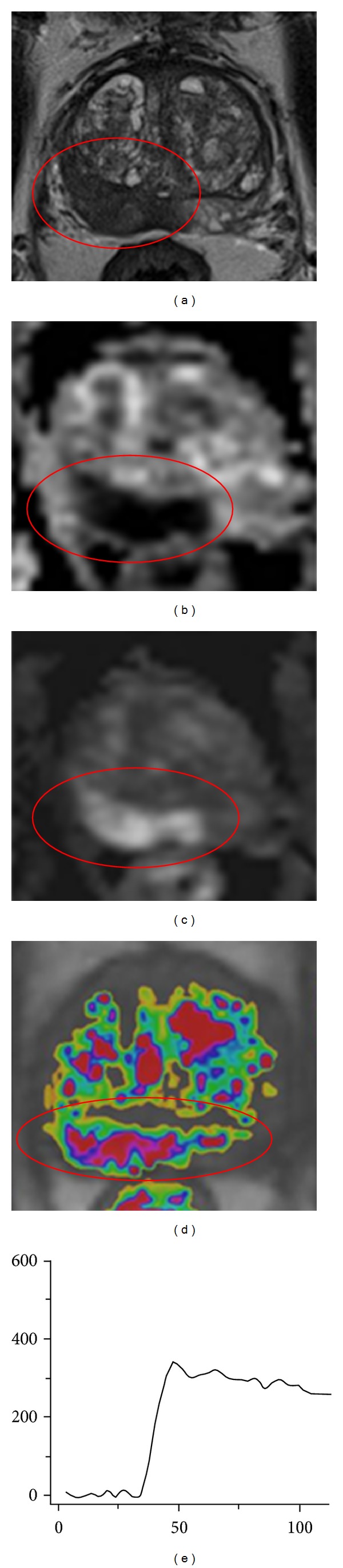
Example of multiparametric MR images of an 83-year-old male, with a PSA level of 10 ng/mL and 0 negative transrectal ultrasound guided biopsy sessions. The encircled lesion scored PI-RADS score 5 on T2W, DWI, and DCE images. Because the lesion is located in the peripheral zone, DWI is the dominant parameter and the final PI-RADS score was PI-RADS 5. (a) Axial T2-weighted image. (b) Axial ADC map. (c) Axial DWI with *b* = 1400. (d) Axial DCE image. (e) Curve of the DCE image.

The multiparametric images of Figure 6 show an example where all parameters show the same PI-RADS score. These images were acquired in an 83-year-old male with a PSA level of 10 ng/mL and no previous biopsy sessions. On the T2-weighted images a large lesion with low signal intensity and broad capsule contact in the peripheral zone is present. This is scored as PI-RADS 5. The same lesion shows hypointense signal on the ADC map and hyperintense signal on the calculated *b*1400 image (PI-RADS 5). DCE shows a curve type 3 with enhancement in an unusual region (PI-RADS 5). For these reasons, the final PI-RADS score is 5 as well. The presence of clinical significant cancer was histopathologically confirmed with MR-guided biopsy, showing a Gleason 5 + 4 = 9.

**Figure 7:**
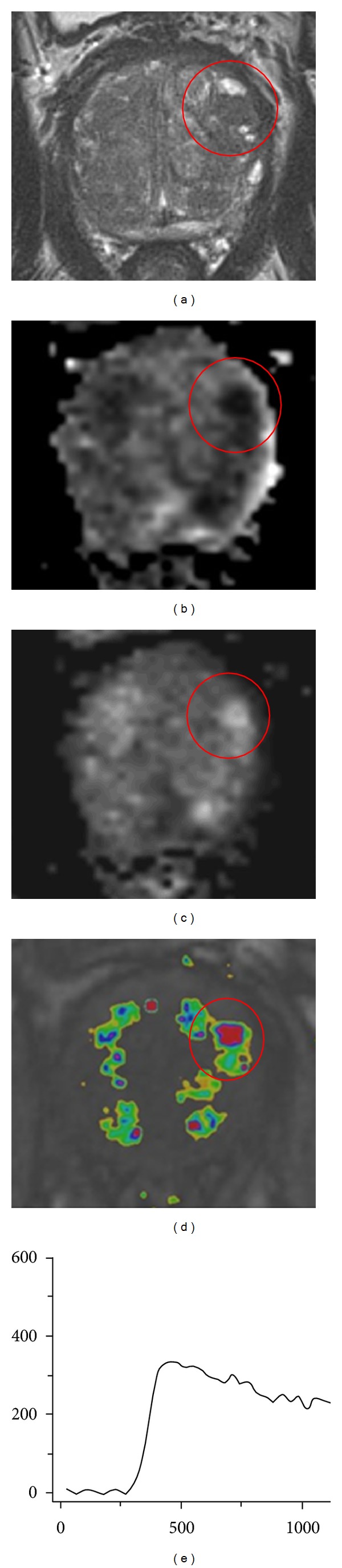
Example of multiparametric MR images of a 68-year-old male, with a PSA level of 40 ng/mL and 3 negative transrectal ultrasound guided biopsy sessions. The encircled lesion scored PI-RADS 2 on T2W image, PI-RADS 5 on DWI, and PI-RADS 4 on DCE. Because the lesion is in the transition zone, T2W is the dominant parameter, and the final PI-RADS score was PI-RADS 2. (a) Axial T2-weighted image. (b) Axial ADC map. (c) Axial DWI with *b* = 1400. (d) Axial DCE image. (e) Curve of the DCE image.

In Figure 7 the multiparametric MR images of a 68-year-old male are shown. This man had a PSA level of 40 ng/mL and had 3 negative transrectal ultrasound guided biopsy sessions. On these images a lesion is seen in the left transition zone. On the T2-weighted images it is scored as PI-RADS 2 because it is a well-marginated area with homogenous low signal intensity originating from a BPH nodule. On the DWI it is scored as PI-RADS 5 because it is a focal area with low signal intensity on the ADC map and high signal intensity on the calculated *b*1400 image. Furthermore, the lesion shows a type 3 enhancement curve, with some focal, however not asymmetric, enhancement. Therefore, it is scored as PI-RADS 4 on the DCE images. Because the individual PI-RADS scores are not concordant, the dominant parameter determines the final PI-RADS score. Since the lesion is located in the transition zone, the dominant parameter is the T2WI and therefore the final PI-RADS score for this lesion is PI-RADS 2. Nevertheless, this patient had a high clinical suspicion for PCa because of his high PSA level and for that reason MR-guided biopsy was performed of this PI-RADS 2 lesion. Histopathological analysis of the biopsy cores confirmed that there was no malignancy but BPH.

In conclusion, the PI-RADS classification is still work in progress and will have further improvement in the future. Furthermore, more studies have to be done to validate the accuracy and interobserver variability.

## Figures and Tables

**Table 1:** PI-RADS scoring system for T2WI—peripheral zone.

PI-RADS score	Criteria
1	Uniform high signal intensity
2	Linear, wedge-shaped, or geographic areas of lower signal intensity, usually not well demarcated
3	Intermediate appearances not in categories 1/2 or 4/5
4	Discrete, homogeneous low signal focus/mass confined to the prostate
5	Discrete, homogeneous low signal intensity focus with extracapsular extension/invasive behavior or mass effect on the capsule (bulging), or broad (>1.5 cm) contact with the surface

**Table 2:** PI-RADS scoring system for T2WI—transition zone.

PI-RADS score	Criteria
1	Heterogeneous transition zone adenoma with well-defined margins: “organized chaos”
2	Areas of more homogeneous low signal intensity, however well-marginated, originating from the transition zone/BPH
3	Intermediate appearances not in categories 1/2 or 4/5
4	Areas of homogeneous low signal intensity, ill defined: “erased charcoal drawing sign”
5	Same as 4, but involving the anterior fibromuscular stroma sometimes extending into the anterior horn of the peripheral zone, usually lenticular or water-drop- shaped

**Table 3:** PI-RADS scoring system for DWI.

PI-RADS score	Criteria
1	No reduction in ADC compared to normal glandular tissue. No increase in signal intensity on any high *b*-value images*
2	Diffuse hypersignal intensity on high *b*-value images* with low ADC; no focal features, linear, triangular, or geographical features, allowed
3	Intermediate appearances not in categories 1/2 or 4/5
4	Focal area(s) of reduced ADC but isointense signal intensity on high *b*-value images*
5	Focal area/mass of hypersignal intensity on the high *b*-value images* with reduced ADC

*(≥*b*800).

**Table 4:** PI-RADS scoring system for DCE.

PI-RADS score	Criteria
1	Type 1 enhancement curve
2	Type 2 enhancement curve
3	Type 3 enhancement curve
+1	For focal enhancing lesion with curve types 2-3
+1	For asymmetric lesion or lesion at an unusual place with curve types 2-3
